# Coronectomy - An alternative approach to remove impacted teeth in oncological patients

**DOI:** 10.4317/jced.55231

**Published:** 2018-10-01

**Authors:** Fabio-Abreu Alves, Marianna-Sampaio Serpa, Wilson Delgado-Azañero, Oslei-Paes de Almeida

**Affiliations:** 1 DDS, PhD, Head of Stomatology Department, A. C. Camargo Cancer Center, São Paulo, Brazil. Professor of the Department of Stomatology, University of São Paulo, School of Dentistry, São Paulo, Brazil; 2 DDS, MSc, PhD student, Department of Stomatology, A. C. Camargo Cancer Center, São Paulo, Brazil; 3 DDS, PhD, Emeritus Professor of Oral Pathology, Universidad Peruana Cayetano Heredia, Lima, Peru; 4 DDS, PhD, Professor of Oral Pathology, Department of Oral Diagnosis, Piracicaba Dental School, University of Campinas, Piracicaba, Brazil

## Abstract

**Background:**

Coronectomy involves the section of the tooth crown leaving the roots in the socket. Possibility of inferior alveolar nerve injury and mandibular fracture are the main indications for this approach. Herein, we describe a case series of coronectomy to highlight its indication in normal and oncological patients.

**Material and Methods:**

A total of 9 patients were submitted to coronectomy, 6 of them were oncological. Three patients were evaluated before head and neck radiotherapy (HNRT), 2 after HNRT and 1 before bone marrow transplantation.

**Results:**

Mean age of the patients was 49 years, most of them male (n=7). Lower third molars were the main teeth that received this procedure, and all cases presented intimate anatomic relationship between the roots and the mandibular canal. Moreover, three cases also presented evident mandibular fracture risk in removing the tooth. During the follow-up period, none complications were observed related to coronectomy and oncological treatment.

**Conclusions:**

Coronectomy is a safe approach including for cancer patients and it should be considered in high-risk impacted teeth extractions.

** Key words:**Coronectomy, impacted teeth, oncological patients, postoperative complications.

## Introduction

Coronectomy or partial odontectomy consists of the removal the crown of a vital tooth leaving the root in the socket (1). This is an alternative procedure developed by Ecuyer and Debien (2), originally indicated for impacted mandibular third molars that are close to the inferior alveolar nerve (IAN). Recently, Samani *et al.* (3), indicated this technique to other teeth, which also present risk of IAN injury and fracture of the jaw.

The indication of coronectomy in oncological patients, to our knowledge, has never been discussed. Patients to be submitted to head and neck radiotherapy (HNRT) and hematopoietic stem cell transplantation (HSCT) need to receive an oral assessment. Dental care before oncological treatments decrease the risks of patients developing adverse effects such as radiation caries, osteoradionecrosis (ORN) and local/systemic infections (4-6). Coronectomy should be an option for these patients particularly if there is a high-risk of damaging the IAN and of mandibular fracture.

The aim of this study was to describe a case series of coronectomy performed in a cancer center. The safety of this procedure for oncological patients is discussed and emphasized.

## Material and Methods

Nine patients submitted to coronectomy were included in this study. Clinical and radiographical information were carefully considered to establish the indication for coronectomy. Moreover, the relationship between the roots and the mandibular canal was classified according to Rood and Shehab’s criteria (7). None of the cases presented associated lesions with the teeth to be removed. All patients received prophylactic amoxicillin for 7 days (500mg, 3 times a day), beginning in the same day of the surgery. Clinical features of the nine cases are described in  Table 1.

**Table 1:**
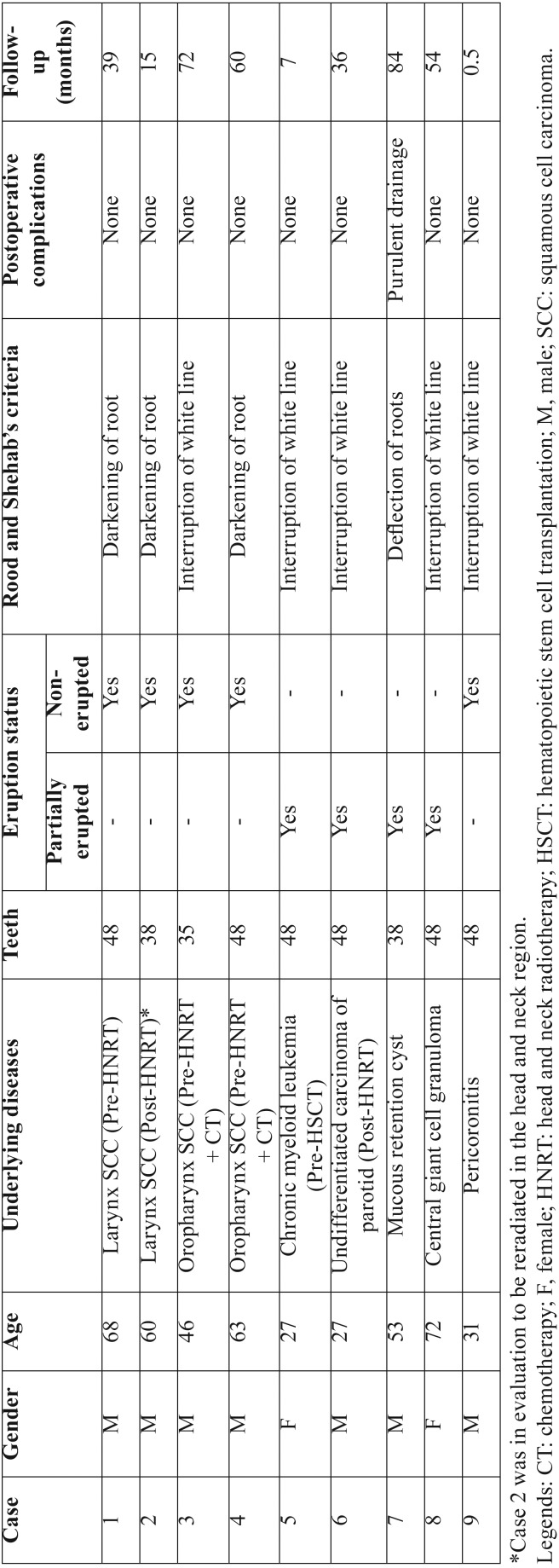
Clinical features of the 9 patients submitted to coronectomy.

We have read the Helsinki declaration and have followed the guidelines in this investigation. In addition, owing to the retrospective nature of our study (case series) and each patient agreed to the consent form, it was granted an exemption by the ethical committee of the A. C. Camargo Cancer Center.

## Results

Of the 9 cases, 6 patients presented a malignant tumor. Five had been diagnosed with squamous cell carcinoma (SCC)¸ 2 of the larynx, 2 of the oropharynx and one of the parotid gland, and one patient had chronic myeloid leukemia. Four out of these 6 patients were referred to the stomatology department for oral evaluation previously to oncological treatment (HNRT and HSCT) and 2 patients (cases 2 and 6) received HNRT 6 and 8 years previously. Case 2, was also in plan to be reradiated. The other 3 patients (cases 7-9) presented benign oral lesions and also impacted third molars ( Table 1).

**Figure 1:**
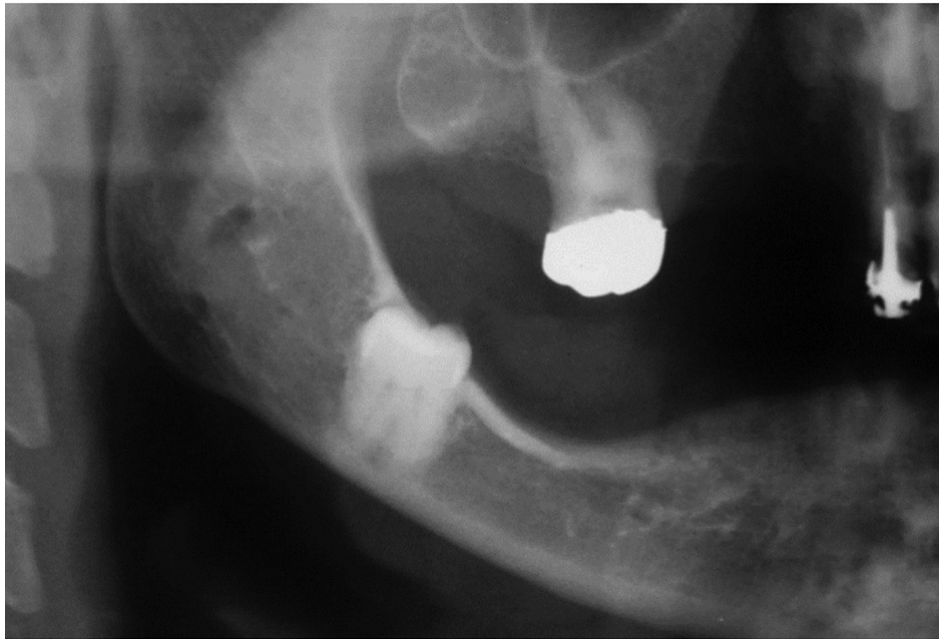
Case 8. Preoperative panoramic radiograph reveals intimate contact of the teeth #48 and inferior alveolar nerve and high-risk of mandibular fracture.

**Figure 2:**
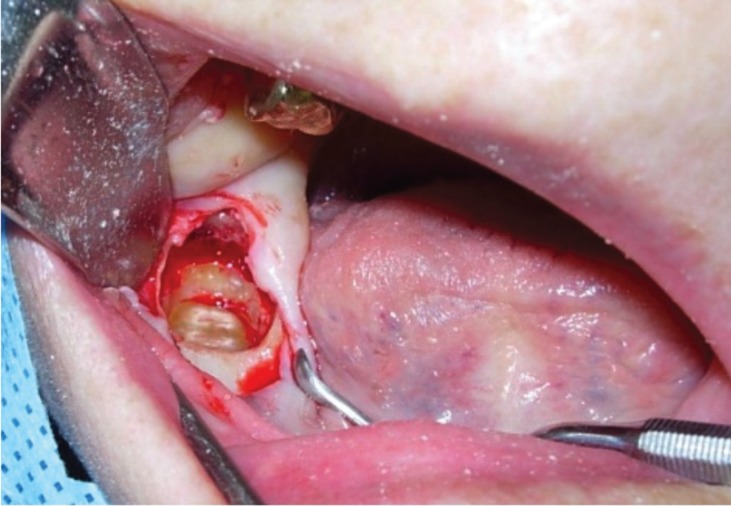
Case 8. Intraoperative accesses after coronectomy.

**Figure 3:**
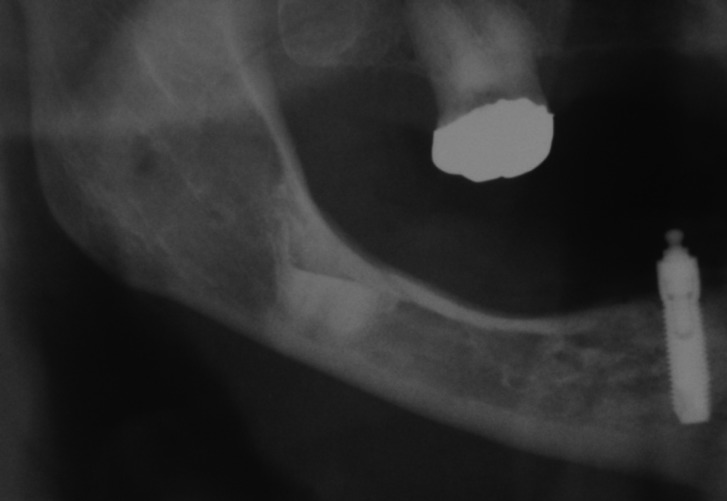
Case 8.Panoramic radiograph taken 3 years later.

Seven patients were male and mean age was of 49 years. The coronectomies were performed in 8 impacted lower third molars and in one impacted lower second premolar. The reasons to choose this alternative surgical approach included the intimate contact between the roots and the IAN, and for cases 1, 3 and 8, the high-risk of mandibular fracture (Figs. 1-3). Apart from that, for the pre-HNRT and HSCT patients, coronectomy was also considered as a safer procedure with fewer chances of postoperative complications that could delay the oncological treatment and for the post-HNRT cases coronectomy was considered a better option to diminish the risk of developing ORN.

There were no intercurrences during the surgeries. Postoperative complications occurred only in case 7, which presented purulent drainage one week after the procedure. The surgical site was irrigated with 0.12% chlorhexidine and clindamycin was administered orally (300mg 4 times a day for 7 days). The healing occurred after 7 days. The mean overall follow-up time was 46 months. Patient 5 died after 7 months due to tumor progress, and patient 9 moved to another state. The other patients remained under follow-up for at least 1 year.

## Discussion

IAN injury occurs in 0.5-0.8% of the mandibular third molars extractions (8). Paresthesia is transitory in most cases; however, it is permanent in 1% of the cases (9). Mandibular fracture occurs trans-operatively in 0.0036% of the lower 3rd. molar surgery and in 0.0046% of the cases as a late complication (10). Although both conditions have low rates, they have severe impact in patient’s quality of life; therefore, coronectomy has been recommended in high-risk dental surgeries (3,11,12). Oncological patients in need of extraction to start radiotherapy are also eligible to undergo coronectomy.

Technically, coronectomy consists of section of the crown at the cemento-enamel junction. The enamel must be removed since it is an inert dental structure of ectodermic origin that prevents the attachment of the gingival connective tissue to its surface, facilitating infections and dry socket formation (13). Instead, the pulp, cement and dentin of the dental root consist of connective tissue that normally is inside the bone. In addition, after coronectomy the cut pulp is cover by a hematic clot, which represents a biological dressing. The roots must be left at 3mm below the crest cortical bone to allow osseous formation over the roots (14,15). The vital remained roots heal without problems (13). Nevertheless, in the cases in which occur infection or present mobility the root have to be extracted completely to avoid unnecessary complications since they may act as foreign bodies (14,16).

The most common complications of coronectomy include root migration, postoperative pain and failed coronectomy (root walk-out during surgery). Surgeons major concern is that leaving the roots will increase the chances of postoperative infection and a consequent need for a second intervention. However, clinical trials and systematic reviews show only few cases present surgical infections, with no significant difference from complete extraction (1,11,17). Leung *et al.* (18) reported that although 2.9% of the coronectomies presented infection in the first week, all cases could be treated with antibiotics and local measures. In the present study, purulent drainage was observed in one case, healing successfully after protocol conduct¸ and no reoperation was needed. Median follow-up was of 3.8 years, and no long-term complications were observed.

Conventional radiographies and/or cone beam computed tomography (CBCT) are necessary to establish coronectomy as the optimal treatment choice. The relationship between the root of the impacted teeth and the IAN have to be properly evaluated. The criteria of Rood and Shehab (7) have been used to identify the cases with higher risk of IAN injury. According to their results, the presence of darkening of the roots, interruption of the white line, and diversion of the mandibular canal are the most reliable signs to predict IAN injuries. In our series, 5 cases presented interruption of white line, 3 darkening of roots and 1 deflection of roots.

Six out of 9 patients of this study had cancer, 3 were evaluated previously HNRT¸ one previously HSCT, and the other 2 had already been submitted to HNRT. For these patients coronectomy was considered as a more secure intervention and presented a lesser risk of complications. In these cases, conventional tooth extraction could eventually cause a postoperative problem, such as an IAN injury or mandibular fracture¸ compromising the oncological treatment. Furthermore, the risk of developing ORN should also be considered¸ mainly in the 2 irradiated patients. All patients were maintained under follow-up and no short or long-term complications were observed.

Although coronectomy is a relatively simple procedure, the lack of experience or guidelines is one of the main causes of failure (15,19). Moreover, Monaco *et al.* (19) observed that surgeons with greater expertise presented lower incidence of complications. Furthermore, it is necessary to explain the patients the advantages and reasons for indicating coronectomy as many are reluctant to accept leaving the roots (15). In our experience, for the success of coronectomy is fundamental that the remnant root must be left completely covered by a mucosal flat to avoid local infection. The present series, to our best knowledge, is the first study to perform such technique in oncological patients. During clinical and radiographic assessment, the surgeon should consider the pros and contras of conventional extraction or coronectomy in order to suggest the best option for the patient, always taking into account that the oncological treatment cannot be altered.

Coronectomy was indicated to avoid IAN injury and mandible fracture, mainly in oncological patients. None complications were observed previously or after HNRT and HSCT. Although a relatively small number of patients included on this series, it can be concluded that coronectomy seems to be a safe and useful procedure in cancer patients.
